# Optimization of Supercritical Fluid Extraction for the Recovery of γ-Oryzanol-Rich Extracts with Improved Bioactivity from Rice Bran

**DOI:** 10.3390/antiox14020206

**Published:** 2025-02-11

**Authors:** João P. Baixinho, Martim Cardeira, Andreia Bento-Silva, Ana Maria Carvalho Partidário, Ana Teresa Serra, Maria do Rosário Bronze, Naiara Fernández

**Affiliations:** 1 iBET, Instituto de Biologia Experimental e Tecnológica, Apartado 12, 2781-901 Oeiras, Portugal; jbaixinho@ibet.pt (J.P.B.); martim.cardeira@ibet.pt (M.C.); tserra@ibet.pt (A.T.S.); mbronze@ibet.pt (M.d.R.B.); 2 Instituto de Tecnologia Química e Biológica António Xavier, Universidade Nova de Lisboa, Av. da República, 2780-157 Oeiras, Portugal; 3 Research Institute for Medicines (iMed.ULisboa), Faculdade de Farmácia da Universidade de Lisboa, Av. das Forças Armadas, 1649-003 Lisboa, Portugal; abentosilva@ff.ulisboa.pt; 4 Instituto Nacional de Investigação Agrária e Veterinária, I.P., Unidade de Tecnologia e Inovação, Av. da República, Quinta do Marquês, 2780-157 Oeiras, Portugal; ana.partidario@iniav.pt

**Keywords:** rice bran oil, supercritical CO_2_, green extraction, antioxidant activity, antiproliferative effect

## Abstract

Rice bran (RB) is a rice processing by-product recognized to be a source of bioactive compounds, including γ-oryzanol and fatty acids, which have interesting bioactivities such as antioxidant and anti-inflammatory effects. This study aims to optimize the supercritical fluid extraction process for recovering these high-value compounds from rice bran with improved bioactivity. A Central Composite Face-Centered Design was employed to optimize the extraction process by varying the temperature (40–80 °C) and pressure (200–500 bar). The optimal extraction conditions were identified at 500 bar and 62 °C that led to the extraction of 17.3% mass yield with 784.5 mg of fatty acids and 36.6 mg of γ-oryzanol per gram of extract, striking a balance between extraction yield and bioactive concentrations. When compared with conventional extractions with n-hexane, supercritical fluid extraction showed similar global yield (18.0 vs. 17.3%) and FA concentration (130.14 vs. 135.70 mg/g of RB) but higher selectivity and extraction yield for γ-oryzanol (18.0 vs. 36.4 mg/g extract; 3.3 vs. 6.3 mg/g of RB). Cellular antioxidant activity assays showed that both extracts reduced the quantity of reactive oxygen species (ROS) up to 50% in Caco-2 cells submitted to oxidative stress. Importantly, supercritical fluid extract was more effective in inhibiting colorectal cancer cell growth (EC50 = 0.9 mg/mL vs. 1.15 mg/mL) than the hexane extract, and this effect was more pronounced than that obtained for pure γ-oryzanol in the same concentration range. These findings highlight the potential of supercritical fluid technology to develop rice bran extracts with antioxidant and antiproliferative properties, underlining the promising applications of this technology in the field of natural product extraction.

## 1. Introduction

Rice (*Oryza sativa* L.) is one of the most important staple food crops consumed globally. In terms of production, rice is the third most important grain in the world behind wheat and corn. Based on rice production quantity data (2000–2020) estimated by the Food and Agriculture Organization of the United Nations (FAO), approximately 757 million tons of rice is harvested worldwide. Historically, rice research has focused on nutrient management and crop genetics, but, since the early 21st century, sustainability has become paramount. Rice processing by-products like bran, husk, and broken rice are often discarded or used as animal feed. The use of these by-products is promoted as part of a circular economy model, promising sustainability and market potential in European agriculture. Among the by-products, rice bran is the most important residue from rice by products, accounting for 0.5 kg of bran and 0.28 kg husk per 1 kg of harvested rice (Rice Knowledge Bank, 2017). Recent studies have reported that rice bran oil (RBO) contains bioactive compounds, such as amino acids, phenolic acids, flavonoids, vitamin E and γ-oryzanol with potential benefit in human health [1,2,3,4,5]. However, despite having a high nutrient value, most by-products of rice production are generally used as an ingredient for animal feed or are not even used.

RBO has been studied for its potential anti-inflammatory properties, attributed in part to its composition on bioactive compounds such as γ-oryzanol, tocopherols, and tocotrienols, which exhibit anti-inflammatory effects. In particular, γ-oryzanol, one of the prominent components of RBO, has shown the ability to reduce inflammation and antioxidant stress [6,7,8,9]. γ-Oryzanol rich extracts, as radical scavengers, possess potent anti-inflammatory activities by inhibiting nuclear factor-kB activation, decreasing oxidative stress gene markers [10]. Another key health-promoting features of RBO is its balanced fatty acid composition. It contains a high proportion of monounsaturated fatty acids (MUFAs), such as oleic acid and palmitoleic acid, and polyunsaturated fatty acids (PUFAs), such as α-linolenic acid and linoleic acid. These MUFAs and PUFAs contribute to reducing low-density lipoprotein (LDL) cholesterol levels, thereby supporting cardiovascular health [4,11].

While Soxhlet and supercritical fluid extraction (SFE) are primarily utilized in laboratory-scale production, solid liquid extraction is the most widely used technique for extracting RBO. Greener extraction techniques have been used recently. The extraction and refining conditions have a significant impact on the concentration of biologically active compounds and the yield of produced oil, making them critical factors for the content of γ-oryzanol and other rice bioactives. Many attempts have been made to find alternative solvents that can increase the overall yield and phytochemical content of RBO while also being more environmentally friendly, even though n-hexane is the preferred solvent, particularly for the large-scale extraction of RBO. The optimal extraction technique should yield a high-purity, fully recovered lipidic fraction free of hazardous chemical residue.

Because γ-oryzanol is a mixture of phytosterols and triterpene alcohols [6] of a predominantly non-polar nature, scCO_2_ is an excellent solvent for γ-oryzanol and a tool to refine RBO. Several studies in the literature reported the advantages of using scCO_2_ for the selective extraction of γ-oryzanol and FA from RB and other rice by-products. The optimization of the extraction conditions, especially temperature, pressure and extraction time, is necessary to maximize extraction efficiency. While Jesus and co-authors identified the optimum extraction conditions for the γ-oryzanol extraction from RBO 200–300 bar under 20 °C [12], other authors demonstrated that high process pressures (689 bar) and temperatures (50 °C or 80 °C) promoted higher global yields and concentrations of γ-oryzanol in RB extracts when compared with other solvents. Therefore, further studies are needed to optimize the selective extraction of γ-oryzanol and FA from RB extracts.

The purpose of this study was to identify the optimum scCO_2_ extraction conditions for the selective extraction of γ-oryzanol and FA from RB, namely, pressure (up to 500 bar) and temperature (from 40 to 80 °C). Furthermore, the impact of scCO_2_ extraction on the bioactivity of the extracts, namely, the antioxidant and antiproliferative effect, was also evaluated and compared with other solvent extraction methods, aiming at highlighting the advantage of this green extraction process to develop bioactive-rich extracts with improved functionalities.

## 2. Materials and Methods

### 2.1. Chemicals

High-pressure extraction studies were conducted using pure grade CO_2_ (99.95%, Air Liquide, Lisbon, Portugal). n-hexane (95%, Carlo Erba Reagents, Val de Reuil, France), water, and ultra-pure water (18.2 MΩ·cm) were among the solvents employed for standard solvent extractions and analyses of the resulting extracts. The latter was acquired using a Millipore-Direct Q3 UV system (Millipore, Burlington, MA, USA). Sigma Aldrich (Taufkirchen, Germany) provided the fluorescein sodium salt, Folin–Ciocalteu’s reagent, Trolox (6-hydroxy-2,5,7,8-tetramethyl chromane-2-carboxylic acid) and AAPH (2,2-azobis(2-methylpropionamidine) dihydrochloride). Phosphate buffer solution (PBS) was prepared using sodium chloride (NaCl), potassium chloride (KCl), sodium phosphate dibasic dihydrate (Na_2_HPO_4_·2H_2_O) from Sigma-Aldrich (St. Quentin Fallavier, France), and potassium phosphate monobasic anhydrous (KH_2_PO_4_) from Amresco (Solon, OH, USA). Gallic acid (PubChem CID: 370) and standard phenolic compounds were also acquired from Sigma-Aldrich (Taufkirchen, Germany).

### 2.2. Raw Material

Milled rice bran from Japônica variety was obtained from Ernesto Morgado S.A (Barra, Portugal), Portugal in February 2021. After reception, the material was placed into plastic bags and stored at −20 °C until additional analyses were conducted. Throughout the experiments, rice bran was protected from light and kept at room temperature within a desiccator.

### 2.3. Extraction Procedures

#### 2.3.1. Supercritical CO_2_ Extractions

An SFE system (Thar Technology, Pittsburgh, PA, USA, model SFE-500F-2-C50) with a 500 mL cylinder extraction cell and two separate separators, each with a 500 mL capacity, and independent temperature and pressure control was used to perform supercritical CO_2_ extractions. Previously, Nunes et al. [13] described this apparatus.

Glass beads were added to the extraction tank along with 20 g of source material to reduce solvent usage. Under the guidance of an automated back pressure regulator (Thar SFC ABPR, Thar Technology, Pittsburgh, PA, USA), the vessel was pressurized with CO_2_ until it reached the necessary pressure (between 100 and 550 bar).

A Thar SFC P-50 high-pressure pump was used to feed liquid CO_2_ into the extraction vessel at a rate of 15 g/min. The CO_2_ was then expanded into the first fraction collector, where the extracts were collected in an ice bath. The extraction continued in a continuous mode for three hours.

##### Experimental Design Analysis/Statistical Analysis

Models of the recovery of FA and γ-oryzanol from japonica rice bran were created using response surface methodology. Through the use of SFE, these bioactive compounds were isolated as a function of temperature and pressure using a Central Composite Face-Centered Design (CCFC). An experimental design is used to obtain as much information as possible about a process from the fewest possible trials. CCFC, which allows for the identification of interactions between variables that are not detectable with traditional methods, was used to test the chosen variables. Following a thorough evaluation of previous research, the variables examined in this study—temperature and pressure—and their range were selected [14,15,16,17,18]. This selection process involved analyzing the methodologies and determining the most relevant variables. This approach not only reinforces the robustness of our study design but also aligns our research with established scientific frameworks.

In order to optimize the temperature and pressure extraction conditions, these variables were coded at three levels, −1, 0 and +1, and experiments were conducted based on a CCFC design (Table 1).

The CCFC data were examined using the MODDE program (MODDE Go 12.1 32-bit, Umetrics, Sweden) to determine the mass yield, γ-oryzanol, and FA concentration in the extracted materials. Each factor’s linear and quadratic impacts, together with their interactions, were computed. For every set of experimental data points, a surface that was described by a second-order polynomial equation was fitted.

#### 2.3.2. Soxhlet Extraction

Solid–liquid extraction was performed to conventionally obtain the RBO containing fatty acids and γ-oryzanol. For Soxhlet extraction, the RB sample (20 g) was placed into cellulose thimbles (22 mm × 80 mm, Whatman (plugged with glass beads to avoid transfer of sample particles in the distillation flask) and placed in Soxhlet apparatus. Solvent n-hexane (400 mL) was added to the flask and boiled for six hours above boiling temperature (69 °C). In order to exclude any solid remnants of the unresolved raw material, the extract was filtered using 125 mm qualitative filter paper from FILTER-LAB and centrifuged using an Eppendorf Centrifuge 5810 R. At 40 °C and with reduced pressure, the extract was dried in a rotary evaporator. Extractions were performed in triplicate.

### 2.4. Chemical Characterization

#### 2.4.1. Analysis of γ-Oryzanol by LC-MS

The LC-MS analysis was performed on the SFE and SOX-Hex extracts using an SCIEX QTRAP 6500+ (Framingham, MA, USA). Chromatographic separation was achieved on an ACQUITY UPLC BEH Shield RP18 Column, 130 Å, 1.7 µm, 2.1 mm × 150 mm (Waters cat. 186003376) at 25 °C. For the mobile phase, ACN:MeOH, 50:50 (*v*/*v*) was used as eluent in an isocratic program.

The 6500+ QTrap was operated in negative ion mode using electrospray ionization (ESI). As a curtain gas (CUR), nebulizing gas (GS1), and drying gas (GS2), nitrogen was employed. The following settings were made for all other instrumental parameters: the drying gas was heated to 650 °C, the ion spray voltage was set at 4300 V, and the CUR was set at 20 psi, GS1 at 50 psi, and GS2 at 35 psi. For every analyte, the mass transitions, entrance potential (EP), exit potential (CXP), collision energy (CE), and declustering potential (DP) were optimized. The Multiple Reaction Monitoring (MRM) mode was used to operate the MS. The software used for data collection and analysis was MultiQuant 3.0.2 and Analyst 1.6.3. The identification of γ-oryzanol was performed on the basis of their retention times compared to the standard (±2%) and the ratio of the first and second transition abundances (within 90–110% of the value obtained for the standard).

#### 2.4.2. Analysis of Fatty Acids by GC

SFE and SOX-Hex extracts obtained were treated in order to convert the existing FA into to the corresponding Fatty Acid Methyl Esters (FAMEs) in a two-step acid-catalyzed method [19], for gas chromatography with flame ionization detector (GC-FID) analysis. Extracts were prepared by acid esterification with BF3 in methanol after a saponification step (methanolic NaOH). After performing a liquid–liquid extraction using isooctane containing BHT (5 µg/mL) as a preservative, the samples were kept at −20 °C and protected from light until they could be examined further. A Thermo Scientific TRACE GC Ultra (Thermo Scientific, Milano, Italy) GC-FID was used to analyze fatty acids. As an internal standard, hexadeca-noic acid (1 mg/mL) was employed. A J&W DB-23 capillary column (Agilent Technologies, Inc., Santa Clara, CA, USA) with an internal diameter of 60 m × 0.25 mm and a phase thickness of 0.25 μm were used to separate the sample components.

### 2.5. Antioxidant Activity—Oxygen Radical Absorbance Capacity (ORAC) Assay

This test evaluated the sample’s antioxidant species’ capacity to prevent fluorescein from being oxidized by peroxyl radicals produced from AAPH. The procedure outlined in Serra et al. (2011) [20] was used to carry out the assay. Briefly, 150 μL of disodium fluorescein (0.3 μM) and 25 μL of extracts were added to each well of a black 96-well microplate. Following a 10 min incubation period, 25 μL of 2,2′-Azobis (2-amidinopropane) dihydrochloride (AAPH) (153 mM) was added to initiate the reaction. A FLx800 fluorescence microplate reader (FL800 Bio-Tek Instruments, Winooski, VT, USA) was used to measure the fluorescence (Ex/Em 485 ± 20/528 ± 20 nm) over the course of 40 min at 37 °C. As a blank, AAPH and FL solutions were made in a 75 mM phosphate-buffer saline (PBS) with a pH of 7.4. The results were presented as Trolox equivalent antioxidant capacity (TEAC) per gramme of extract (μmol TEAC/g extract), with Trolox serving as the reference standard. Using up to three distinct dilutions, the samples (SFE-opt and SOX-Hex extracts) were examined in triplicate.

### 2.6. Cell-Based Assays

#### 2.6.1. Cell Culture

The American Type Culture Collection (ATCC, USA) and the Deutsche Sammlung von Mikroorganismen und Zellkulturen (Braunschweig, Germany) provided the human colon cancer cell lines HT29 and Caco-2, respectively. With the addition of 10% (*v*/*v*) heat-inactivated fetal bovine serum (FBS), 1% (*v*/*v*) non-essential amino acids (NEAAs), and 1% (*v*/*v*) penicillin–streptomycin, the Caco-2 cell line was cultivated in Dulbecco’s Modified Eagle Medium (DMEM). Roswell Park Memorial Institute medium (RPMI) supplemented with 10% FBS was used to cultivate HT29 cells. In 75 cm2 culture flasks, the cells were regularly maintained as monolayers at 37 °C in a humidified environment consisting of 95% air and 5% CO_2_.

#### 2.6.2. Cytotoxicity Assays

Cytotoxicity assays were performed using confluent and non-differentiated Caco-2 cells. This cell model is widely used as it shares some characteristics with crypt enterocytes, considered an acceptable intestinal epithelium model [21]. Briefly, cells were seeded at a density of 2 × 10^4^ cells/well into 96-well plates, and they were left to grow for seven days, with a medium renewal occurring every 48 h. On the seventh day, cells were cultured using diluted SFE-opt and SOX-Hex extracts in a culture medium that was enhanced with 0.5% FBS and 1% NEAA. The concentrations of the extracts that were tested ranged from 0.04 to 5 mg/mL. The control group consisted of cells cultured solely with the culture medium. According to the manufacturer’s instructions, cells were rinsed once with PBS (Sigma-Aldrich, St. Louis, MO, USA) after 24 h, and their viability was evaluated using the CellTiter 96^®^ AQueous One Solution Cell Proliferation Assay (Promega, Madison, WI, USA) with MTS reagent. A BioTek EPOCH2 Microplate Reader (BioTek, Winooski, VT, USA) was used to measure the absorbance at 490 nm, and the percentage of living cells in comparison to the control was used to express cell viability. Three separate tests were carried out in triplicate.

#### 2.6.3. Antiproliferative Activity

HT29 cell lines, a popular model for in vitro colorectal cancer research, were used to assess the extracts’ antiproliferative effects [20,22]. Cells were cultivated in 96-well culture plates at a density of 1 × 10^4^ cells/well in the RPMI with 10% FBS added, to put it briefly. Cells were incubated with SFE-opt and SOX-Hex extracts diluted in culture medium (DMEM + 0.5% FBS) at concentrations ranging from 0.04 to 5 mg/mL following a 24 h culture. As a control, cells cultured solely with a culture medium were used. As previously stated, the MTS reagent was used to measure cell proliferation. In comparison to the control, the percentage of living cells was used to express cell viability. In triplicate, three independent experiments were conducted. Dose–response curves were used to determine the half maximal effective concentrate ions (EC50), which were then expressed as milligrams of extract per milliliter.

#### 2.6.4. Cellular Antioxidant Activity

With a few minor adjustments, a dichloro-dihydro-fluorescein di-acetate (DCFH-DA) probe was used to measure the production of intracellular ROS [23,24].

In 96-well plates, Caco-2 cells were seeded at a density of 2 × 10^4^ cells/well in the DMEM + 10% FBS, 1% NEAA, and 1% PenStrep. The medium was thrown away three to four days after seeding, and PBS was used to wash the cells. Cells were co-incubated with 25 µM of DCFH-DA in PBS for 1 h at 37 °C with 5% CO_2_ after being treated with specific concentrations of SFE-opt and SOX-Hex extracts (1.25, 2.5, and 5 mg/mL). Furthermore, tests were conducted on γ-oryzanol at the same concentration range found in rice bran extracts (44, 87, and 174 µg/mL). The positive control was quercetin at 20 µM. After washing the cells with PBS, 2,2′-Azobis(2-amidinopropane) dihydrochloride (AAPH) was administered at a non-cytotoxic concentration of 600 µM, and the cells were then incubated at 37 °C. Using a Microplate Fluorimeter FLx800 (Biotek Instruments, Winooski, VT, USA) with excitation and emission wavelengths of 485 ± 20 nm and 528 ± 20 nm, respectively, fluorescence was recorded after one hour. The percentage of fluorescence intensity compared to the control (cells treated with DCFH-DA alone and oxidant (600 µM AAPH)) was used to express the results. Three separate tests were carried out in duplicate for every sample concentration.

#### 2.6.5. Statistical Analysis

The mean value ± SD, derived from a minimum of three separate experiments, was used to express the results. The results were statistically analyzed using GraphPad Prism 9 (GraphPad Software, Inc., La Jolla, CA, USA). The results were analyzed using one-way analysis of variance (ANOVA) and the Tukey test for multiple comparisons after a normal distribution and homogeneous variance of the data were confirmed. To ascertain whether the means were significantly different in the case of heterogeneous variances, a suitable unpaired Student’s *t*-test was conducted. In every instance, a *p*-value of less than 0.05 was deemed statistically significant.

## 3. Results and Discussion

### 3.1. Optimization of Bioactive Extraction from RB Using Supercritical CO_2_

Previous studies showed that SFE is a promising technology to obtain γ-oryzanol and FA-rich extracts from rice bran. Bitencourt et al. [15] assessed the ability of scCO_2_ to extract γ-oryzanol from rice bran. Its solubility was measured at various temperatures and pressures, showing a solubility from 0.13 to 1.57 g γ-oryzanol/kg CO_2_. A crossover pressure at 300 bar was observed where solubility behavior changes with temperature. Below the crossover pressure, the solubility decreased with increasing temperature and above, the solubility increased with increasing temperature. An extraction at 60 °C and 400 bar, was also performed, resulting in the recovery of 22.2% oil with 1.55% γ-oryzanol in its composition. The presence of oil reduces the solubility of γ-oryzanol, indicating a higher concentration of the compound in the final oil or raffinate fractions. Karin Tomita et al. [16] also revealed that γ-oryzanol concentrations in the oil obtained by scCO_2_ extraction at different temperatures were significantly varied, with crossover pressure at 300 bar.

In our work, the extraction yield and bioactives profile of RBO extract were investigated. A preliminary evaluation was necessary to set up an extraction procedure of bioactives, namely, FA and γ-oryzanol, from rice bran. The yield of extracted oil from rice bran using a CO_2_ apparatus (continuous mode at 40 °C and 500 bar during 3 h) was 15% *w*/*w* (on dry weight basis). Results showed that oleic acid (C18:1) (40.4%) and linoleic (C18:2) (36.3%) were the most important fatty acids in the oil, followed by palmitic acid (17.2%) and that about 78% of the total fatty acids in RBO were unsaturated fatty acids. The unsaponifiable constituents of RBO mainly comprised γ-oryzanol, tocotrienols, and tocopherols. Characterization of the initial SFE extracts revealed a concentration of 24.9 mg γ-oryzanol/g extract.

**Figure 1:**
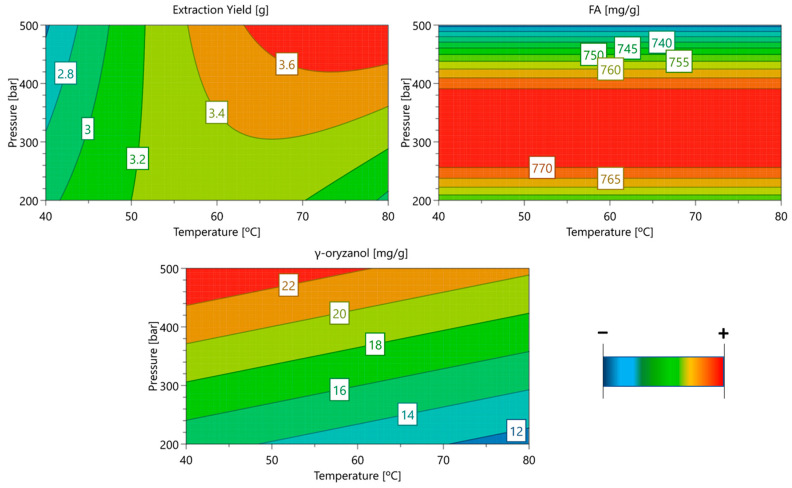
Response surfaces fitted to bioactive compound concentration in the extract as a function of temperature and pressure. The concentration (mg/g extract) of each compound is displayed in white squares based on the three axes’ conditions. The minimum extraction for each SL is shown in blue, and the maximum extraction is shown in red.

To maximize the quality of the RBO, increasing the γ-oryzanol percentage in oil, a design of experiments (DOE) was performed to optimize the extraction selectivity for γ-oryzanol. The effect of process variables on the extraction yield, γ-oryzanol and FA concentrations in the extract was evaluated on a Central Composite Face-Centered Design with two independent variables: temperature and pressure. The obtained results are shown in Table 2, and the respective response surfaces fitted for those bioactive compounds extraction are represented in Figure 1.

The data’s regression analysis revealed that, for a 95% confidence level (*p* < 0.05), the extraction pressure had a positive impact on γ-oryzanol concentration in the final extracts (*p* = 0.002). The same parameter had also a significantly quadratic effect on FA extraction selectivity (*p* = 0.019), indicating that the middle of the experimental range is where the ideal pressure levels are found. Both pressure and temperature (*p* = 0.006) had a positive impact on the extraction yield.

Correlating with the model’s projected outcomes, the maximum extraction yield was obtained (18.3%) when the highest pressure and temperature were used (80 °C, 500 bar). At these conditions, high density CO_2_ (0.88 g/mL) was used as extraction solvent. The lowest extraction yield value (2.51%) was obtained when experiments were conducted at highest pressure but low temperatures (40 °C). 

Regarding the γ-oryzanol concentration in the final RB extracts, the effect of the pressure is visible when comparing extractions conducted at low and high pressures. At the same temperature (80 °C), the extraction performed at 500 bar (22.72 mg/g extract) resulted in an extraction of 2.5 times more selective for γ-oryzanol compared to those performed at 200 bar (8.94 mg/g extract).

The selectivity for the FA extraction was entirely impacted by the pressure chosen, and there was no significant effect of temperature. The highest value achieved was 787.22 mg/g of extract, operating at medium pressures (350 bar).

The yield of RBO was shown to increase with pressure (Figure 1).

In the experimental domain studied, at 420 bar, 55 °C, with a constant flow rate of 15 gCO_2_/min flow, the ideal conditions to optimize all responses (extraction yield, γ-oryzanol and FA concentration) were reached. However, as the FA concentration in the RBO was comparable for all the conditions, we were able to perform a further adjustment to promote the extraction of γ-oryzanol rather than FA. The optimal conditions were 500 bar and 62 °C to increase the extraction yield and γ-oryzanol concentration in the final extract. The overall concentration of this bioactive was enhanced by increasing the pressure. This optimization (SFE-opt) resulted in a 17.3% extraction yield with 784.5 mg FA/g extract and 36.6 mg of γ-oryzanol/g extract in its composition, resulting in a total recovery of 6.33 mg γ-oryzanol/g RB (Table 3).

SFE performance was compared to conventional solid–liquid extraction using a Soxhlet apparatus with n-hexane as solvent (SOX-Hex) to extract these bioactive compounds from rice bran. SOX-Hex exhibited an 18% extraction yield and the bioactive amount extracted was lower than when using the SFE-opt condition (130 mg FA/g of RB) and 3.3 g γ-Oryzanol/g of RB). Similar extraction yields and FA content were obtained for both approaches, indicating the affinity of these compounds by non-polar solvents. However, the selectivity of scCO_2_ for γ-Oryzanol was approximately two times higher (SFE-opt).

**Figure 2:**
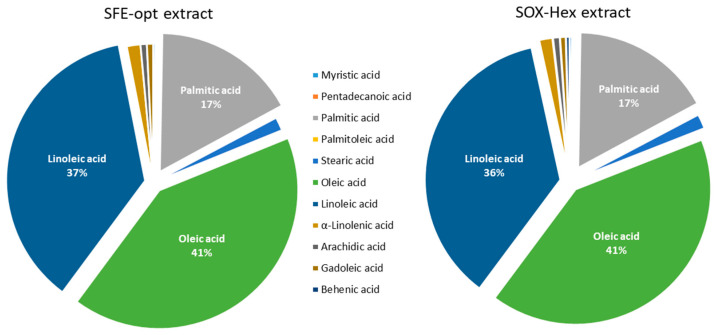
Fatty acid profile of RBO obtained by SFE (SEF-opt) and Soxhlet (SOX-Hex).

The FA composition of SFE-opt is shown in Figure 2. The RBO extracted by SOX-Hex was found to be richer in total FA and the proportion of PUFA and MUFA were comparable. These PUFA are reported to contribute to reducing low-density lipoprotein (LDL) cholesterol levels, thereby supporting cardiovascular health [25]. The higher differences were seen in the oleic acid (C18:1) and linoleic acid (C18:2) contents, as they appear in a higher amount in the SFE-opt. Our results demonstrate that scCO_2_ was consistently superior to organic solvent extraction in recovering γ-oryzanol from rice bran. The increased pressure and temperature conditions used significantly enhance the extraction yield compared to traditional methods. The variation in conditions (e.g., temperature, pressure, time) between studies leads to different outcomes, but it is clear that higher pressures (500 bar) and optimized temperatures (around 60 °C) consistently result in higher yields of γ-oryzanol. Compared to already published studies, scCO_2_ revealed to be the best choice for rice bran valorization. The final SFE-opt extract obtained was superior in FA and γ-oryzanol concentration when compared to other solvents and extraction methods. This finding is consistent with Xu and Godber’s [26] work. According to their research, among the organic solvents examined, a solvent combination of 50% hexane and 50% isopropanol (*v*/*v*) at 60 °C for 45–60 min generated the maximum yield (1.68 mg γ-oryzanol/g RB). Nevertheless, they were able to obtain a yield of 5.39 mg of γ-oryzanol/g of RB in SFE at 50 °C and 690 bar pressure for 25 min, which was almost four times higher than the greatest yield of solvent extraction. In a study conducted by P. Sookwong et al. [17], the authors aimed to simultaneously quantify vitamin E, γ-oryzanol, and xanthophylls from RB using supercritical CO_2_ extraction. The optimized conditions used were 60 min, 43 °C, 374 bar with 10% ethanol as a modifier. The greatest amount of γ-oryzanol (2.73 mg/g RB) was extracted from HPY non-glutinous red rice.

Unlike the study conducted by Sookwong et al., which used a lower pressure of 374 bar, our use of 500 bar likely enhanced the extraction efficiency by increasing the density of CO_2_, improving its solvating power for γ-oryzanol. In addition to higher γ-oryzanol recovery, our extract demonstrated significant enrichment in monounsaturated and polyunsaturated fatty acids, further highlighting the value of our optimized conditions for rice bran valorization.

Similar to our study, Perretti et al. [27] concentrated on improving the value of rice bran through SFE. This study likely explored the optimization of SFE processes to efficiently recover valuable compounds at high pressures. The highest amount of γ-oryzanol (18.0 mg/g of extract) was obtained at 700 bar and 80 °C. By focusing on the recovery of a specific compound, Jesus et al. [12] achieved the maximum global yield (39 *w*/*w*) with scCO_2_ at 300 bar and 30 °C with the maximum γ-oryzanol recovery rate (31.3 mg/RB extract), relatively high γ-oryzanol content (3.2 mg/g of extract), and a significant presence of monounsaturated and polyunsaturated fatty acids. While the SFE conditions reported by Perretti et al. were effective in maximizing γ-oryzanol yield, the higher pressures and temperatures used may not compensate for the final selectivity of the process. Jesus et al. achieved a relatively high global yield (39% *w*/*w*) with scCO_2_, but the γ-oryzanol content (3.2 mg/g of extract) was lower compared to our results. This suggests a trade-off between maximizing total yield and concentrating specific bioactive compounds.

These findings highlight the importance of fine-tuning extraction conditions, particularly pressure, temperature, and modifier usage, to overcome the lack of balance observed in previous studies. By addressing these challenges, our approach achieved not only higher γ-oryzanol yields but also a more cost-effective and scalable solution for rice bran valorization. Our final results, under optimized conditions (500 bar and 62 °C), SFE-opt reached a yield of 17.3%, with a concentration of 784.5 µg FA/mg of extract and 36.6 mg and 6.33 mg γ-oryzanol/g of extract and RB, respectively.

### 3.2. Bioactivity Evaluation of the RBO

#### 3.2.1. Antioxidant Activity

Different techniques have been developed to evaluate the effectiveness of dietary antioxidants, whether they are present as pure substances or in food extracts. These techniques focus on several processes of the antioxidant defense system, such as the scavenging of lipid peroxyl radicals, the suppression of lipid peroxidation, and the chelation of metal ions [28]. The oxygen radical absorbance capacity (ORAC) assay is one of the in vitro techniques frequently used to gauge the antioxidant capacity of food components and is based on the reaction of peroxyl radicals produced by the decomposition of AAPH at 37 °C, with a fluorescent probe (fluorescein) to form a non-fluorescent product; however, in the presence of an antioxidant, the decay of the probe is inhibited because the antioxidant competes for peroxyl radicals. Therefore, this method was selected to compare the antioxidant value of two extracts, namely SOX-Hex and SFE-opt extracts. In addition, a cellular antioxidant activity assay (CAA), using a human intestinal cell line (Caco-2) was used to better understand the reactive oxygen species (ROS) scavenging capacity of extracts, because some processes related to the uptake, distribution, or metabolism of bioactive compounds are better addressed than in chemical techniques [29]. The selection of the cell model—confluent Caco-2 cell culture—was because it mimics the human intestinal epithelium, as it shares some characteristics with crypt enterocytes, as it is considered a valid intestinal model and has been widely implemented to assess the effect of chemical and food compounds on intestinal function [21,30].

**Figure 3:**
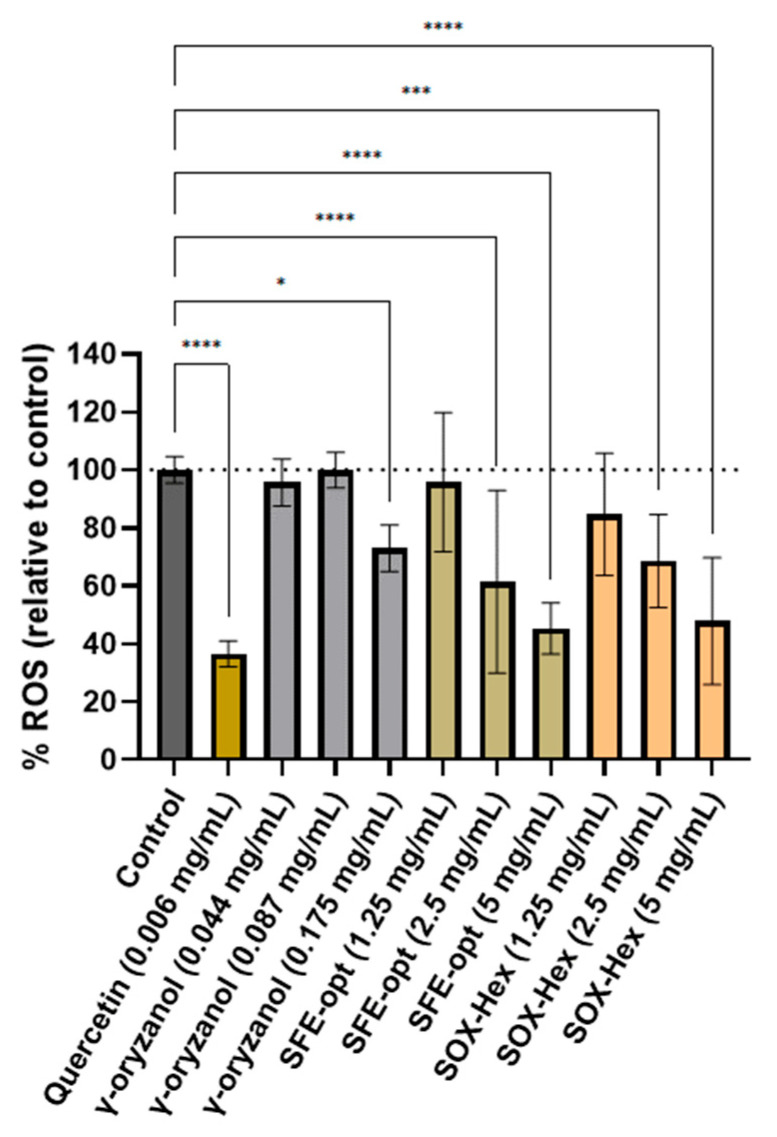
The cellular antioxidant capacity of each extract and γ-oryzanol, expressed as a percentage of ROS compared to the control. The mean ROS percentage in relation to the control ± SD is used to express the results. Significant differences from the control are indicated by the symbol *; * *p*-value ≤ 0.05, *** *p*-value ≤ 0.001, and **** *p*-value ≤ 0.0001.

Results showed that both extracts, SOX-Hex and SFE-opt ORAC values 480 ± 50 μmol of TEAC/g and 450 ± 48 μmol of TEAC/g, respectively, are similar (*p*-value > 0.05). These results can be related with the similar composition of samples. A similar result was observed in the CAA assay, where non-toxic concentrations of both extracts (1.25, 2.5 and 5 mg/mL) presented equivalent capacity in scavenging ROS generated by AAPH at a cellular level (Figure 3). In general, both extracts inhibited ROS formation (from 100% to 42%) in dose-dependent effect (Figure 3). Importantly, when compared with γ-oryzanol (at the same concentration range present in the extracts: 0.044–0.175 mg/mL), both extracts presented higher antioxidant capacity, suggesting that other compounds present in the samples may be scavenging ROS at a cellular level, thus helping to protect the cells from oxidative stress. Additionally, these extracts (at 5 mg/mL) showed similar cellular antioxidant effects as quercetin (0.006 mg/mL), a bioactive flavonoid known for its considerable cellular antioxidant activity [31,32].

These data are in accordance with the literature. Tyagi, A. et al. [33] demonstrated that ethanolic rice extracts show dose-dependent cellular antioxidant activity with increasing concentrations from 0.5 mg/mL to 5 mg/mL in the Caco-2 cell model, showing a higher reduction in the ROS percentage. In another cell line, Saji et al. [1] showed that the incubation of rice bran extracts at 0.025–0.25 mg/mL for 6 h reduced the generation of intracellular ROS in RAW264.7 cells stressed with H_2_O_2_.

#### 3.2.2. Antiproliferative Effect

**Figure 4:**
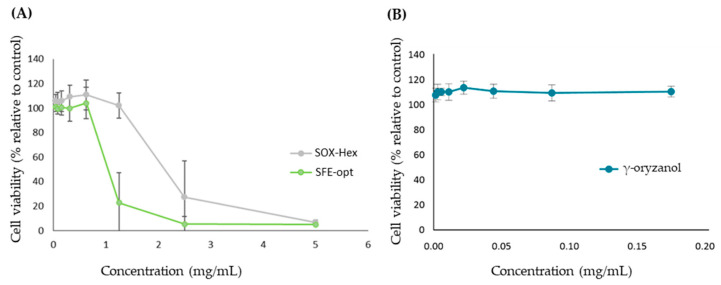
Antiproliferative effect in HT29 after 24h incubation of (**A**) RB extracts, namely SOX-Hex and SFE-opt, and (**B**) γ-oryzanol at the same maximum concentration range present in the extracts.

The antiproliferative effect of the samples was evaluated using a human colorectal adenocarcinoma cell line (HT29). This cell line was subjected to treatment with non-cytotoxic concentrations of extracts (from 0.04 to 5 mg/mL, previously determined) for 24 h. For both extracts, dose–response curves (Figure 4) were performed, and the concentration that decreases 50% of cell viability (effective concentration—EC50) was calculated (Table 4).

Both SOX-Hex and SFE-opt extracts inhibited HT29 cell proliferation in a dose-dependent manner (Figure 4) with the SFE-opt extract inducing a higher antiproliferative effect. This effect could be explained by the presence of other bioactive compounds rather than oryzanol, since, for the same concentration range, the standard compound alone did not show antiproliferative effects.

The antiproliferative effect of rice bran has already been reported by other authors. Ghasemzadeh, A. et al. [34] studied the antiproliferative properties of black, red, and brown RBO in breast cancer cell lines (MCF-7 and MDA-MB-231) and obtained EC50 values ranging from 119 to 382 mg/mL. The extracts produced in our work presented a higher antiproliferative effect due to the lower EC50 value (about 100 to 200 times lower, Table 4), but this result could be explained by the difference between the cell lines.

Our results suggest that the extracts developed in this work, mainly the rice bran extract developed by the optimized SFE, have improved bioactive effect, regarding the capacity in inhibiting cancer cell growth. More studies are needed to identify the compounds responsible for this effect in SFE extracts as well as to understand the mechanism of action of the antiproliferative effect.

## 4. Conclusions

Rice bran is a promising source of an oil, rich in valuable lipophilic compounds, FA, and γ-oryzanol.

Under optimized conditions (500 bar and 62 °C), SFE (SFE-opt) yielded 17.3% extraction with a concentration of 784.5 µg FA/mg of extract and 36.6 mg γ-oryzanol/g of extract. These results are comparable to conventional Soxhlet extraction using hexane (18% extraction yield, 723.4 µg FA/mg of extract, and 32.7 mg γ-oryzanol/g of extract), showcasing SFE as a sustainable alternative to traditional solvent-based extraction methods for obtaining RBO, particularly in the recovery of γ-oryzanol.

The cell-based assays provided valuable insights into the bioactivity of the extracts, revealing their antioxidant and antiproliferative effects.

Overall, this study reinforces the use of SFE as a valuable tool for recovering high-value compounds from rice by-products, emphasizing its advantages as a toxic-solvent-free, faster alternative to conventional methods. Future studies should include a life cycle analysis (LCA) to compare both methodologies, offering a more comprehensive evaluation of their environmental impacts and overall sustainability.

## Figures and Tables

**Table 1:** Variables used in the design of experiments (DOE). In the context of process optimization involving two process variables, temperature and pressure, the utilized variables are those within the DOE, while the observed values correspond to the coded representations of these variables.

Exp No.	Temperature (°C)	Pressure (bar)	Temperature Coded	Pressure Coded
1	40	200	−1	−1
2	80	200	+1	−1
3	40	500	−1	+1
4	80	500	+1	+1
5	40	350	−1	0
6	80	350	+1	0
7	60	200	0	−1
8	60	500	0	+1
9	60	350	0	0
10	60	350	0	0
11	60	350	0	0

**Table 2:** Conditions and results of the DOE of the SFE.

Exp No.	Temperature (°C)	Pressure (Bar)	Extraction Yield (%)	Total FA	γ-Oryzanol
				(mg/g Extract)	
1	40	200	15.1%	757.64	14.49
2	80	200	14.7%	772.31	8.94
3	40	500	12.6%	716.35	24.89
4	80	500	18.2%	707.41	22.72
5	40	350	13.8%	752.64	20.62
6	80	350	17.6%	787.22	17.56
7	60	200	15.9%	723.50	15.4
8	60	500	18.3%	746.41	18.78
9	60	350	16.8%	784.49	15.38
10	60	350	15.8%	782.52	16.75
11	60	350	16.7%	745.21	19.41

**Table 3:** Results obtained with the optimized conditions (500 bar, 62 °C) of the SFE (SFE-opt). The standard deviation values are calculated based on the results of the triplicates of the DOE.

Extraction Yield (%)	Total FA (mg/g Extract)	Total FA (mg FA/g RB)	γ-Oryzanol (mg/g Extract)	γ-Oryzanol (mg/g RB)
17.3 ± 0.5	784.4 ± 18.4	135.7 ± 3.9	36.4 ± 3.5	6.3 ± 0.7

**Table 4:** EC50 values obtained for all the extracts in HT29 cells, with 24 h incubation. EC50 values are expressed as mean ± SD. The results identified with different letters (a to b) in the same column are statistically different (*p*-value ≤ 0.05).

Extract	Antiproliferative Effect—EC50 (mg/mL)
SFE-opt	0.9 ± 0.04 ^a^
SOX-Hex	1.5 ± 0.19 ^b^

## Data Availability

Data are contained within the article.
